# A potential presence of cryptic species in the Asian common dark firefly *Lucidina biplagiata*

**DOI:** 10.1186/s40851-026-00273-8

**Published:** 2026-09-04

**Authors:** Yuichi Oba, Noriomi Fujimori, Hidehiko Hirata

**Affiliations:** Department of Environmental Biology, Chubu University, Kasugai, 487-8501 Japan

**Keywords:** Cryptic species, Firefly, Japan, Taxonomy

## Abstract

The presence of two morphological forms in the Asian dark firefly *Lucidina biplagiata* (the Japanese name: Oba-botaru) in Japan was suggested by Dr. Sakyo Kanda in 1935, but they have long been considered to be the morphotypes of a single species. In this study, we examined the genetic variations and temporal emergence of Oba-botaru specimens at a single locality in Aichi, Japan. Phylogenetic analyses of mitochondrial and nuclear markers revealed two distinct lineages within *L. biplagiata*. Adult emergence patterns differed between the two sympatric groups. We found that the mode of the pronotal color patterns could be largely distinguished between the two groups, and also between bimodal emergence. Preliminary mating experiments showed that males and females from the two genetic groups did not mate in captivity. These results strongly support the existence of two sympatric, reproductively isolated species-level lineages within *L. biplagiata*.

**Supplementary Information:**

The online version contains supplementary material available at https://doi.org/10.1186/s40851-026-00273-8.

## Background

Currently, over 2,600 valid extant species are recognized in the family Lampyridae [1], of which 51 species are distributed in Japan [2–6]. Their life cycles and behaviors have also been well-studied; however, most of the research focuses on the ‘lightningbug firefly’ [7] species, which show bright on-and-off flash bioluminescence in the adult stage, such as the Genji firefly *Nipponoluciola cruciata*, Heike firefly *Aquatica lateralis*, and Hime firefly *Luciola parvula*. They are nocturnal and use flash signals for sexual communication [8–13]. In contrast, the ‘dark firefly’ [7] species, which are non-luminous or emit very weakly, have been comparatively less studied. The genus *Lucidina* is a typical ‘dark firefly’ group, currently comprising 17 species in the world, of which 5 species are in Japan: *Lucidina accensa*, *Lucidina biplagiata*, *Lucidina natsumiae*, *Lucidina okadai*, and *Lucidina yakushimensis* [1, 2, 5]. They emit strongly in larval stages but are practically non-luminescent in the adult stage. Instead, they are diurnal and probably use pheromones for sexual communication [9–11].

*Lucidina biplagiata* (Motschulsky, 1866) is one of the most common and widely distributed firefly species in Japan, found in Hokkaido, Honshu, Shikoku, and Kyushu, as well as in Sakhalin and the Kurils Islands [2, 14]. Historically, this species was first described as *Lychnuris biplagiata* Motschulsky, 1866 from ‘Japon’ [15], then as three different species names, *Lucidota angusticollis* Kiesenwetter, 1874, *Lucidota tabida* Kiesenwetter, 1874, and *Lucidota vulnerata* Kiesenwetter, 1874 from Nagasaki, Yokuhama, and Hyogo or Nagasaki of Japan, respectively [16], and then, *Pyractonema puerile* Olivier, 1902 from Tokyo, Japan [17]; finally, they were included in a single species, *Lucidina biplagiata* [2, 17]. Until recently, this species was also considered to be in Korea [2], but more recent studies have reclassified the Korean *Lucidina* as a new species, *Lucidina kotbandia* Park & Chang, 2005 [18]. The species name *biplagiata* probably means ’two patches’ or ’obliques’ representing the two red spots on the black pronotum.

**Fig. 1:**
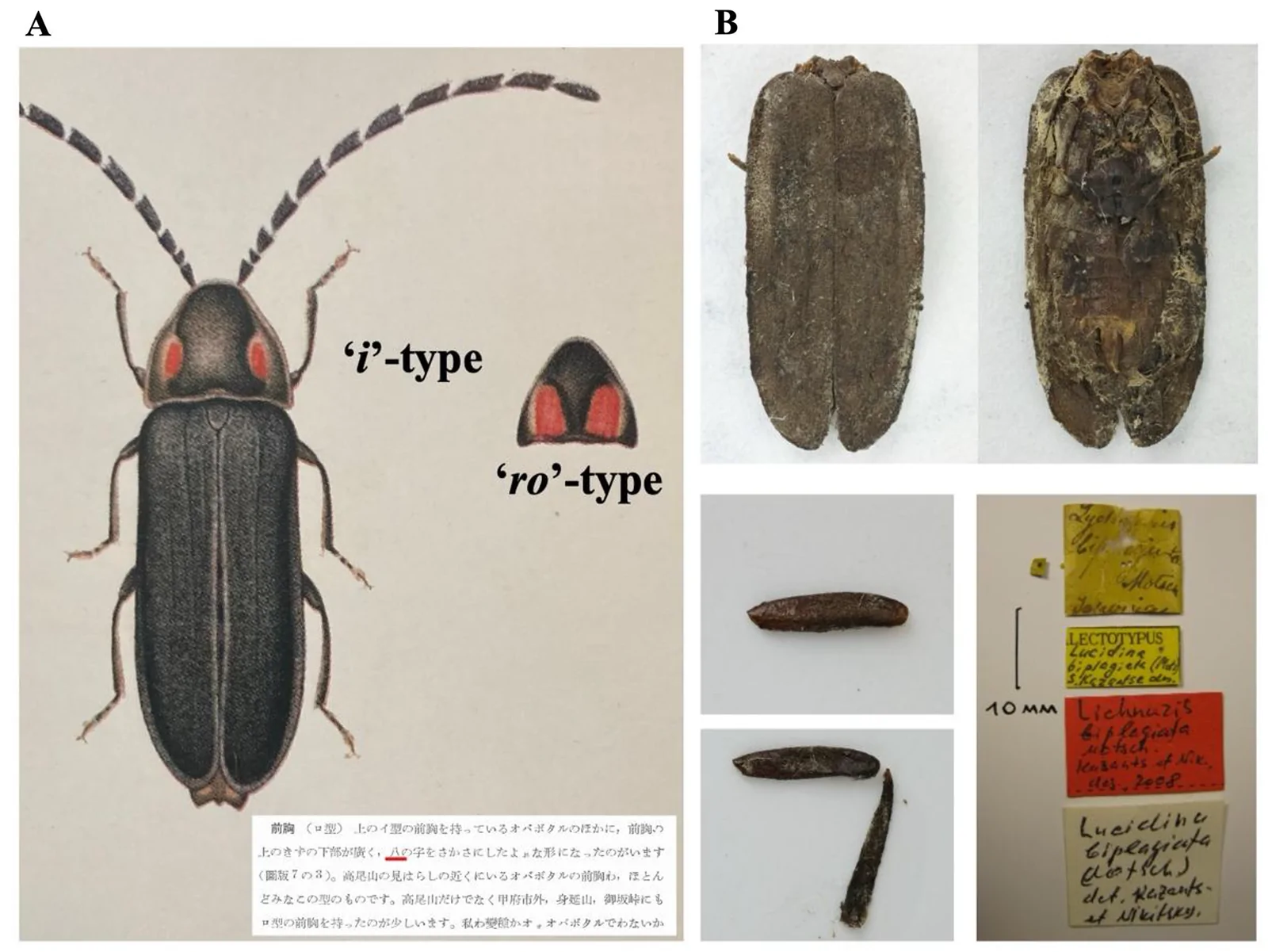
The illustration and description of ‘*i*’-type (male) and ‘*ro*’-type of the adult Oba-botaru in the book by Kanda, 1935 (**A**). The red underline indicates the Japanese character for eight. The lectotype specimen and the labels (**B**). Upper left, whole dorsal view. Upper right, whole ventral view. Bottom left, left mesofemur and left metafemur + metatibia. Bottom right, labels

A legendary firefly researcher, Sakyo Kanda (1874–1939), published a Japanese book entitled “Hotaru” [19], which means ‘the fireflies’, important for the ecology and taxonomy of Japanese fireflies. It is interesting to note that in this book, he pointed out the presence of two distinct morphotypes of *L. biplagiata* in Japan based on the shape of red patches on the pronotum (Fig. 1A); he called them ‘*i*’-type and ‘*ro*’-type (‘*i*’ and ‘*ro*’ represent the first and second characters of the old Japanese alphabet). The red patches are oval-shaped in the ‘*i*’-type, while the patches in the ‘*ro*’-type are wider at the posterior, “like the reversed shape of the Japanese Kanji character for eight” (Fig. 1A) [19]. However, no other researchers had ever taken note of this point.

In Aichi Prefecture, where our home is located (Supplemental Fig. S1), two *Lucidina* species, *L. biplagiata* (the Japanese name, Oba-botaru) and *L. accensa* (Ô-oba-botaru), are distributed. In the neighboring prefectures of Gifu and Mie, *Lucidina okadai* (Kokuro-oba-botaru) is also found. The morphological differences between these three species are distinct [11]. In this study, to examine the presence of cryptic species of *L. biplagiata* sympatrically, we examine the temporal emergence, genetic variation, and preliminary mating trials of the specimens collected in an area of Kota Town in Aichi Prefecture, mainly focusing on the shape of two red patches on the pronotum, which is inspired by Kanda’s suggestion. We found that the specimens collected in the Kota population were separated into two distinct genetic types. During our analyses, we also recognized that the temporal emergence is bimodal but overlapping. Additionally, the shapes of the two red patches are gradual. To evaluate whether the two genetic types were separable by the combination of the two factors, we conducted a two-dimensional analysis of the emergence timing and the inner angle of the two red patches.

## Methods

### Field observation and specimen collection

The observation of the natural habitat and specimen collection were systematically conducted in the woodland located on the east side of the reservoir Ôi-ike Pond at Ôkusa, Kota Town, Aichi Prefecture, Japan (N34.9 E137.2, Alt. 100–110 m) from May to August in 2019–2021 (Supplemental Fig. S1). The specimens were typically found on the grass and lower leaves beside the path in the morning, and we collected them manually (Supplemental Table S1). The specimens for DNA and morphological analyses were freshly immersed in the 99.5% ethanol to minimize DNA degradation and morphological deformation.

### DNA analysis

**Fig. 2:**
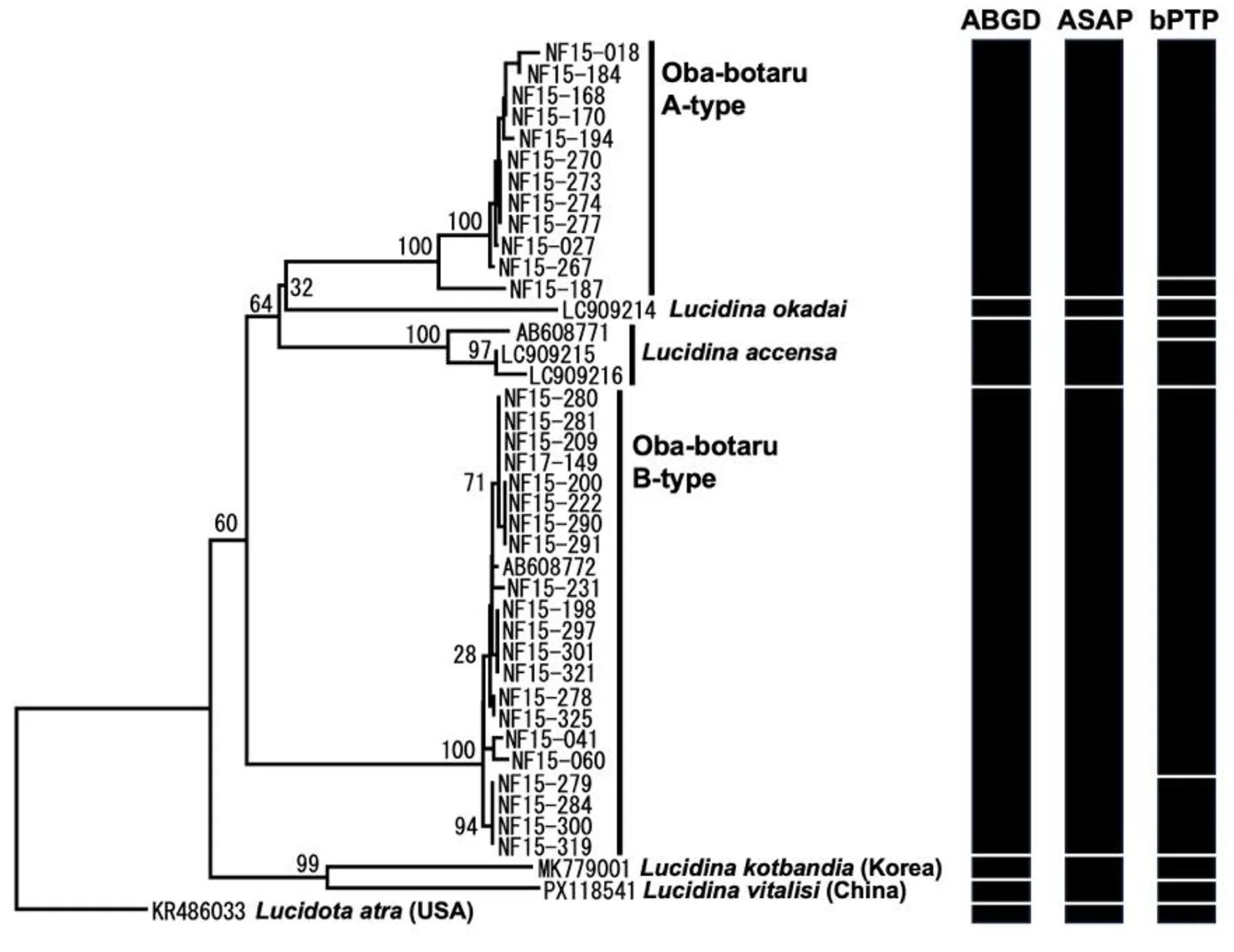
Phylogenetic tree of Oba-botaru in Kota Town and its relatives. Nucleotide sequences of 657 bp from *COI* fragments were used for the analysis. The numbers on the nodes represent the bootstrap value (%) by 1,000 replicates. Black boxes indicate the corresponding result for each species delimitation method

DNA was extracted using DNeasy Blood & Tissue Kits (Qiagen, Hilden, Germany) from 1 to 2 legs of the specimen stored in 99.5% ethanol. PCR was performed to amplify the barcoding region of the cytochrome *c* oxidase subunit I (*COI*) using a primer set LCO1490 and HCO2198 [20] and the fragment of 28 S ribosomal DNA (*28SrDNA*) using a primer set 28 S-F1 and 28SR-01 [21]. The PCR product was directly sequenced at Macrogen (Tokyo, Japan), and the sequences were deposited in the GenBank/ENA/DDBJ database (accession numbers; LC913026 - LC913066). Multiple alignment and tree reconstruction (NJ) were performed using MAFFT ver. 7 (https://mafft.cbrc.jp/alignment/server/) with default settings (Jukes-Cantor). Molecular species delimitation analyses based on *COI* sequences were conducted using three complementary methods: Automatic Barcode Gap Discovery (ABGD) [22], Assemble Species by Automatic Partitioning (ASAP) [23], and the Bayesian implementation of the Poisson Tree Processes model (bPTP) [24]. ABGD and ASAP analyses were performed using the Spart explorer server (https://spartexplorer.mnhn.fr/delimitation, accessed on 21 Aug, 2026), with default parameters. The bPTP analysis was performed using the bPTP web server (https://species.h-its.org, accessed on 11 Aug, 2026), with 100,000 MCMC generations and all other parameters set to default. The ML tree inferred from the *COI* dataset was used as the input tree for the bPTP analysis (Fig. 2).

### Morphological analysis

The specimen preserved in 99.5% ethanol was taken out into the air and photographed using a microscopic digital camera MDCE-5 C, which was equipped with the image software ScopeImage9.0 (Novel, Ningbo, China). The pronotum was positioned flat for accurate measurement. The inner angle (degree) of two red patches on the pronotum was measured with a protractor by drawing auxiliary lines at the boundary between the red patch and black bump on the printed image of the pronotum. To ensure measurement repeatability, all specimens were measured by a single individual. The standard deviation of the average angle among 16 independent measurements was < 10% for A-type and < 30% for B-type specimens (standard deviation for low-angled specimens is inevitably larger). Because the middle part of the red patches is nearly straight, the measurements are expected to be highly reproducible. Additionally, the ratio of the lengths between the antenna and elytra was accurately measured using the printed image.

### Mating experiments

**Fig. 3:**
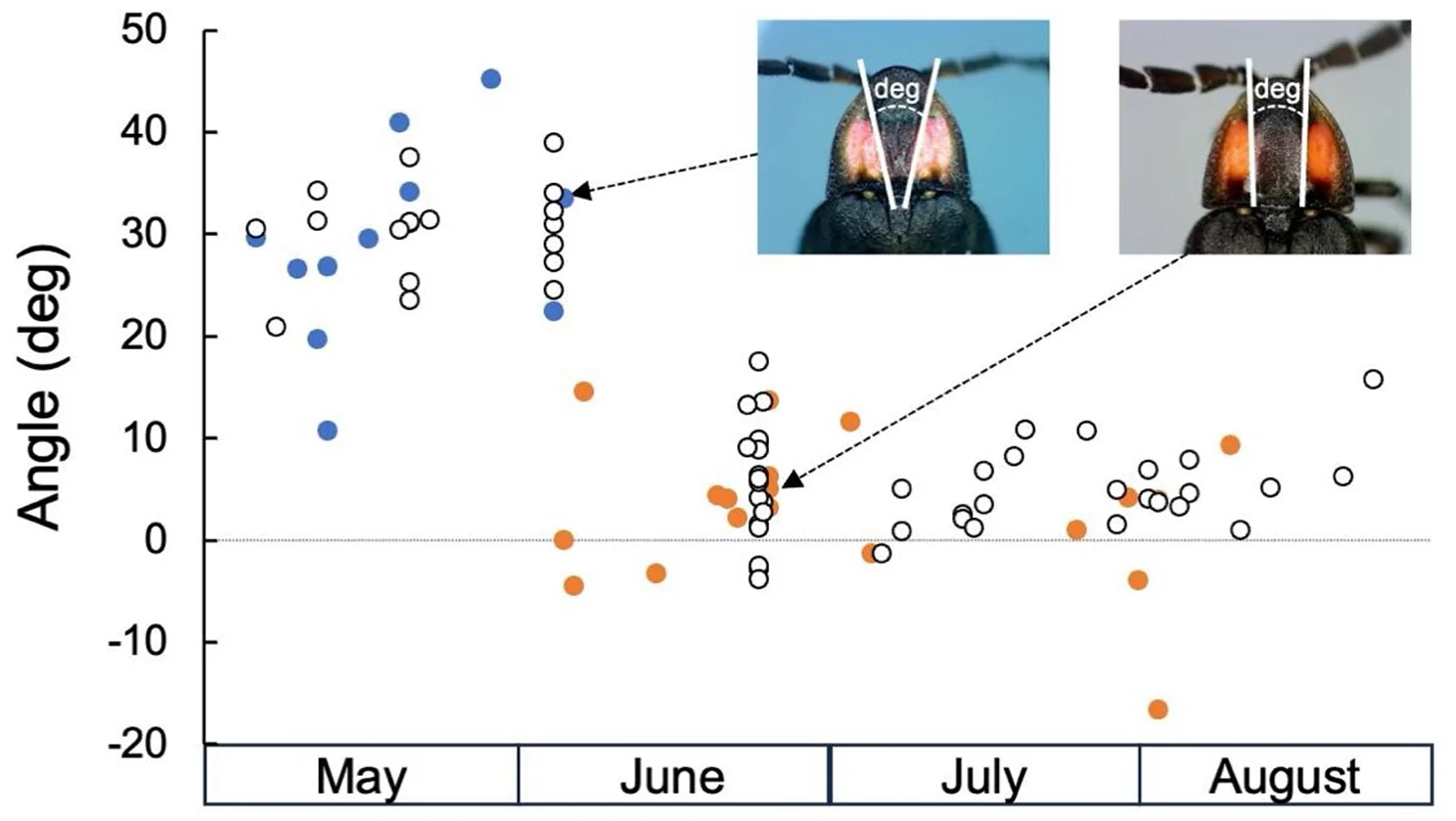
The Collection date and the inner angle (deg) of two red patches on the pronotum of the male specimens (inset). Colored circles represent the specimens whose genetic type was determined: Blue circle, A-type, Orange circle, B-type. Open circles represent the specimens whose genetic type was not determined

A pair of living male and female specimens collected at Kota Town (Ôkusa, Alt. 100–110 m), Toyota City (Ôtaga, Alt. 750 m), or Shitara Town (Damine, Alt. 750 m), all in Aichi Prefecture, were transferred into a translucent container (230 ⋅ 80 ⋅ 100 mm) with a lid. Mating behavior was observed during the daytime. The type of the specimens, A-type or B-type, was judged by their collection date (Fig. 3) and/or genetic analysis (Fig. 2), while specimens that were questionable as to their type, whether A-type or B-type, from collection date and/or genetic analysis were excluded from these experiments. We considered the male and female to have ‘mated’ when the male mounted on the female (mating success is ‘success’). Mating behavior was observed within 20 min for the same genetic type pair. When the male did not mount on the female within 2 hours, we considered them as ‘unmated’ (mating success is ‘failed’).

## Results

### Bimodal temporal emergence in adults

Adult individuals resting on leaves along the survey path were counted visually. The observed sex ratio was approximately 10:1 (male: female). Emergence showed a bimodal seasonal pattern from May to August every year (Supplemental Fig. S2). The result suggested the presence of temporal isolation between two groups.

### Genetic differences

The phylogenetic tree of mitochondrial *COI* genes revealed two distinct lineages in *L. biplagiata*, which we tentatively named A-type and B-type (Fig. 2). These findings indicate that the Kota population of *L. biplagiata* is genetically divided into two groups. This distinction is further supported by nuclear *28SrDNA* analysis (Supplemental Fig. S3). Molecular species delimitation analyses were performed using *COI* sequences, showing that ABGD, ASAP, and bPTP all support the species differences of A-type and B-type (Fig. 2).

### Relationships between pronotal morphology and temporal emergence

Since the specimens we used for the morphological analyses were uniformly prepared by fixing fresh living samples in 99.5% ethanol, the morphological deformation of them is considered to be minimal in comparison to the air-dried specimens, which were made under uneven humid conditions and sometimes the body parts were shrunk. We conducted combined analyses of emergence timing and inner angles of two pronotal red patches in male specimens, revealing a correlation with genetic types (Fig. 3; Supplemental Figs. S4, S5); every year, larger angled (around 30 degrees) specimens appeared earlier, and smaller angled (around 5 degrees) specimens appeared later. They are associated with genetic A-type and B-type, respectively. However, these criteria are not definitive: for example, specimen NF15-187 had smaller angled patches (10.7 degrees) but appeared seasonally earlier, and the genetic type was A; also, both A-type and B-type specimens were collected on the same day, 5 June, in 2021 (see Supplemental Table S1). In females, these angles do not correlate with emergence timing, suggesting that this criterion cannot be applied to females. Male *L. biplagiata* has elongated antennae to detect female pheromones. The ratio of the length of male antennae to the length of elytra, as an indicator of body size, showed statistical differences between groups (Welch’s *t*-test, *p* < 0.001), representing longer antennae in B-type than those in A-type in general (Supplemental Fig. S6).

### Cross-mating compatibility

Due to the temporal emergence being separated between two genetic types, it was challenging to prepare both types simultaneously for the cross-mating test. However, by collecting specimens from higher altitude localities in Shitara and Toyota, which are cooler than Kota, we successfully conducted two independent heterotypic mating experiments. In those cooler areas, the emergence timing of the two genetic types is probably different from Kota. The results of the cross-mating experiments showed that mating between specimens of the same genetic type was 100% successful, while mating between different genetic types was 100% unsuccessful (Supplemental Table S2). These results are still limited but indicate that the female attracted the same type of male but did not attract a different type of male, suggesting the presence of strong premating isolation between these two types.

## Discussion

Both mitochondrial and nuclear markers consistently support the existence of two distinct lineages within the traditional concept of *L. biplagiata*. However, the relationships among these lineages and other *Lucidina* species remain unresolved. Mating experiments are still limited but support the lack of reproductive compatibility between the two lineages. These results strongly support the existence of two reproductively isolated species-level lineages. All molecular species delimitation analyses performed based on *COI* sequences supported the species differences of the two lineages, but the judgment should be suspended because we did not analyze the specimens from the whole distribution range. As the ‘dark firefly’ group uses pheromones for sexual communication as mentioned above, it is predicted that their pheromone components are different between the two genetic types, named A-type and B-type, and function as an ecological mechanism maintaining reproductive barriers. They can be roughly distinguished by a combination of two factors, their emergence timing and the inner angle of the pronotal red patches; A-type species have larger angled patches and appear earlier, while B-type species have smaller angled patches and appear later. Remarkably, the possibility of two forms within *L. biplagiata* was proposed by Kanda (1935) nearly ninety years ago, but has remained largely untested until the present study. Judging by the text and illustration in his book, Kanda’s ‘*i*’-type and ‘*ro*’-type [19] probably correspond to the present B-type and A-type, respectively. Ohba reported the unusually long emergence, as for a Japanese firefly species, of *L. biplagiata* (June to August) in some regions [11], and we expect that he observed the emergence of the two genetic types which we found in this study together.

One of the authors (YO) had the opportunity to examine the lectotype specimen of *L. biplagiata* [25] at the Zoological Museum of the Moscow Lomonosov State University, Moscow, Russia. The specimen was a male, but lacked a head, antennae, some legs, and pronotum (Fig. 1B). Due to these missing parts, we currently lack a method to determine the inner angle of the red patches on the lectotype specimen, making it difficult to ascertain which type is the true *L. biplagiata* and which is a new species. However, if Motschulsky’s naming of ‘biplagiata’ [15] implied two-obliques, similar to the reversed Japanese character for eight (‘*ro*’-type in [19]), our A-type might represent the true *L. biplagiata*. Further study is essential if the taxonomic status of these two types is to be determined. In particular, it will be important to conduct detailed morphometric analyses of parts other than the pronotum using specimens from various localities and compare them with photographs of the lectotype specimen.

## Conclusions

This study aimed to determine whether cryptic species of the Asian dark firefly *L. biplagiata* coexist sympatrically by combining analyses of molecular phylogeny, phenology, pronotal color pattern, and antennal size, alongside conducting limited cross-mating tests. The obtained data were consistent with the view that currently recognized *L. biplagiata* comprises two distinct species-level lineages.

## Supplementary Information

Below is the link to the electronic supplementary material.

Supplementary Material 1

Supplementary Material 2

## Data Availability

Supplementary material is available online.
