# Supplementary material for: A potential presence of cryptic species in the Asian common dark firefly Lucidina biplagiata
Source: Zoological Lett. 2026 Sep 4;12:14. doi: 10.1186/s40851-026-00273-8 (PMC13563847; doi:10.1186/s40851-026-00273-8)
Supplement: Supplementary file 1 [file 40851_2026_273_MOESM2_ESM.docx]

**A potential presence of cryptic species in the Asian common dark firefly *Lucidina biplagiata***

Yuichi Oba^*,1^, Noriomi Fujimori^1^, Hidehiko Hirata^1^

Department of Environmental Biology, Chubu University, Kasugai 487-8501, Japan

* Corresponding author: yoba@fsc.chubu.ac.jp ORCID 0000-0003-2108-4947

^1^ These authors contributed equally to this work.

**SUPPLEMENTARY TABLE and FIGURES**

**(Table S1 is not included in this file)**

**Table S2.** Mating experiments

| Entry | Experiment (2021) | Male × Female^a^ | Mating success |
| --- | --- | --- | --- |
| 1 | 30 - 31, May | **A** (29 May, Kota) × **A** (29 May, Kota) | Success |
| 2 | 25 - 26, June | **A** (20 June, Shitara) × **B** (25 June, Kota) | Fail |
| 3 | 25, June - 1, July | **B** (25 June, Kota) × **B** (25 June, Kota) | Success |
| 4 | 18 - 19, July | **A** (10 July, Toyota) × B (17 July, Kota) | Fail |
| 5 | 18, July | B (17 July, Kota) × B (17 July, Kota) | Success |
| 6 | 31, July | **B** (31 July, Kota) × B (31 July, Kota) | Success |

^a^A, A-type specimen, B, B-type specimen. Bold A/B indicates specimens with genetic types determined by *COI* sequence analysis and collection date. While, regular A/B indicates those determined by collection date alone. The collection date and locality are shown in parentheses.

**Fig. S1.** Research field at the eastside of Ôi-ike Pond, Kota Town, Aichi Prefecture, which is close to the boundary of Okazaki City. The paths for the specimens' survey are shown in orange marking. Google Map.

**Fig. S2.** Temporal emergence of the adult Oba-botaru from May 1 to August 31. Open, gray, and black circles represent the counts of specimen numbers in 2019, 2020, and 2021, respectively.

**Fig. S3.** Phylogenetic tree of Oba-botaru in Kota Town and its relatives. Nucleotide sequences of 424 bp from *28SrDNA* fragments were used for the analysis. The numbers on the nodes represent the bootstrap value (%) by 1,000 replicates.

**Fig. S4.** Photographs of the pronotum in genetic A-type and B-type. Specimen ID numbers in blue and red represent the male and female, respectively. The scale bar is shown in the last picture (1 mm).

**Fig. S5.** Collection date and inner angle (deg) of two red patches on the pronotum of male specimens in 2019 (A), 2020 (B), 2021 (C), and female specimens in 2019-2025 (D). Colored circles represent the specimens for which the genetic type was determined: Blue circle, A-type; Orange circle, B-type. Open circles represent the specimens for which the genetic type was not determined.

**Fig. S6.** Ratio distribution of the antenna length to elytra length in the male specimens collected before 4 June (blue) or after 24 June (orange). The vertical value indicates the number of the specimens measured.
